# Graduate Study on the Continent

**Published:** 1908-09-05

**Authors:** 


### GRADUATE STUDY ON THE CONTINENT.

*France.*—Most of the hospitals are open to foreign graduates on payment of special fees. For an ordinary visit or attendance at a demonstration the production of the visitor's card is usually quite sufficient. The following hospitals have special lectures and classes, particulars of which may be obtained on application to the respective Deans :—Paris : Hôtel-Dieu, 560 beds ; La Pitié, Charité, Necker, Lariboisière, Beaujon, Laennec, Andral, all general hospitals varying from 300 to 800 beds ; Saint-Louis, Midi, Lourcine, Clinique, Maison de Santé, Trousseau, Pasteur, special hospitals with from 100 to 250 beds. Excellent courses are given at some of the provincial centres in France. Caen and Lille, both university towns, attract many English students. Orleans and Marseilles possess good hospitals where foreign graduates are allowed to study.

*Germany.*—All the German Universities extend their cordial hospitality to graduates of sister universities and to diplomates of learned societies. At most of the better-known centres special classes are held for foreign students: these classes are essentially "post-graduate" classes; they are practical and sometimes include laboratory work. Several articles dealing with post-graduate study in Germany have appeared recently in the columns of The Hospital, and to them we would refer any reader interested in the subject.

*Austria.*—Vienna, Prague, and Innsbruck cater for the American and English graduate by advertising special classes in otology, ophthalmology, laryngology, and allied subjects during the vacation. These vacation courses are held at the various special clinics, by some well-known expert who usually lectures and demonstrates in English and allows students to do practical work under him.

*Italy.*—The foreign student is always welcome at every university and in every hospital. At Milan the unrivalled Ospedale Maggiori well repays a few weeks' study. At Rome, Turin, Florence, and Venice there are excellent hospitals and periodical courses of lectures on certain subjects, which may be attended on production of evidence of registration or on payment of a fee.

*Switzerland.*—The best centres are Basle, Zurich, and Berne, at each of which vacation courses are held.

*Holland.*—No facilities are extended to the graduate, but, as in Italy, he is everywhere courteously made welcome. The best clinical teaching is to be found in Amsterdam, Leyden, and Groningen.

*Russia.*—At St. Petersburg alone does the foreign student obtain special facilities for such study, but there he enjoys unrivalled opportunities for studying special subjects. The teaching in surgery, midwifery, and orthopædics is specially good in Russian centres.

